# Evaluation of upper limb superficial venous percussion as a sign of anatomical location and venous permeability. A comparative study of superficial venous percussion to ultrasound findings on non-renal patients and on chronic kidney disease patients

**DOI:** 10.1371/journal.pone.0224825

**Published:** 2019-11-11

**Authors:** Pedro Coelho N. Diógenes, Aline Naiara Azevedo da Silva, Fausto Pierdoná Guzen, Marco Aurelio de Moura Freire, José Rodolfo Lopes de Paiva Cavalcanti

**Affiliations:** 1 Anatomy Laboratory, Medical School, Department of Biomedical Sciences, State University of Rio Grande do Norte, Mossoró, Rio Grande do Norte, Brazil; 2 Nova Esperança College, Mossoró, Rio Grande do Norte, Brazil; University Magna Graecia of Catanzaro, ITALY

## Abstract

The objective of this study was to evaluate venous percussion in the upper limbs as a possible low-cost clinical sign that may contribute to physical examination of the superficial venous system and be useful in the evaluation for punctures and also for the construction of fistulas.

**Methods:**

An analytical cross-sectional study with 70 individuals divided into two groups. Group A consisted of 35 volunteers who were being preoperatively prepared for the construction of arteriovenous fistula. Group B consisted of 35 non-renal patients selected by convenience. Each participant underwent physical examination, including venous percussion, of the dominant upper limb and then ultrasound. Interobserver agreement was assessed between a trained vascular surgeon performing percussion and fourth-year medical student. Accuracy, sensitivity, specificity, positive predictive value and negative predictive value of percussion were determined in relation to ultrasound. The agreement between the methods, venous percussion and venous duplex ultrasound was also evaluated by the Kappa index.

**Results:**

The overall interobserver agreement for the percussion was 0.74 (95% CI 0.632 to 0.851). It was observed that the results were more favorable in the cephalic vein than in the basilic vein, emphasizing that the cephalic is more used in venous punctures, because of its anatomical location and visibility, and in fistula construction. The 35 percussions of the cephalic forearm vein in Group A resulted in a sensitivity of 1.0 (95% CI 0.63 to 1.00), specificity of 0.96 (95% CI 0.81 to 1.00), a positive predictive value of 0.89(95% CI 0.52 to 1.00) and a negative predictive value of 1.00 (95% CI 0.87 to 1.00), with an accuracy of 0.97 (95% CI 0.85 to 1.00) and Kappa index of 0.92 (95% CI 0.77 to 1.00) in relation to ultrasound. Overall, when all venous segments were analyzed in group A, the Kappa index of agreement between the percussion and the ultrasonography reached 0.56 (95% CI 0.401 to 0.72). All venous segments in Group A had a sensitivity of 0.54 (95% CI 0.37 to 0.70) and a specificity of 0.96 (95% CI 0.90 to 0.99). When all venous segments were analyzed in group B, the Kappa index of agreement between the percussion and the ultrasonography reached 0.48 (95% CI 0.34 to 0.62). All venous segments in Group B had a sensitivity of 0.70 (95% CI 0.59 to 0.79) and a specificity of 0.82 (95% CI 0.69 to 0.91).

**Conclusion:**

Venous percussion of the upper limbs has a high positive predictive value and high specificity, when compared to ultrasound as a way to evaluate the patency and adequacy of the cephalic vein. Although there is not enough evidence to preclude ultrasound, percussion should definitely be included in the traditional physical exam evaluation of upper limbs superficial veins.

## Introduction

Peripheral venous punctures are referred to as common procedures, even though few studies have been conducted researching risk factors for difficult venous punctures [1–3]. In emergency situations, even for an experienced professional, venous puncture can be a challenging procedure with anatomic and body type factors affecting its success rates [1,2,4]. Unsuccessful venous punctures can promote a vicious cycle as an unsuccessful attempt leads to increased pain scores and possible phobia for further attempts, increasing puncture difficulty [3,5]. Some guidelines even limit the number of attempts by a single professional in order to avoid emotional exhaustion and reduce iatrogenic lesions[5,6]. About 8% of adults and 14% of children are considered difficult peripheral venous puncture patients[5]. In France, for example, it is estimated that 25 million peripheral venous catheters are placed annually[7]. Even though peripheral venous puncture is a common procedure in clinical practice, and subject to difficulty, few studies have been conducted to identify factors for its success[2].

Out of all vascular access options for hemodialysis, native arteriovenous fistulas constructed with the anastomosis of upper limb superficial veins and arteries are considered the best option[8–11]. Over 300,000 patients depend on a vascular access for hemodialysis[8]. A reduction in morbidity and mortality is expected when a patient is using a native arteriovenous fistula[12], so every effort should be made to construct a native arteriovenous fistula in the prevalent group of patients who depend on hemodialysis[8–11]. On the National Kidney Foundation Kidney Disease Outcome Quality Initiative (NKF KDOQI)^TM^ Vascular Access Working Group document, the preoperative evaluations needed in order to construct a native fistula includes a clinical history, physical examination and arterial and venous duplex ultrasound. In the physical examination, there is no mention for superficial venous percussion analysis as a means to identify venous segments adequate for the construction of a native fistula[8–11].

A preoperative duplex scan as the means to locate the best suitable superficial venous segment for native arteriovenous fistula construction has been analyzed through systematic review and been accepted as the preferred method[13]. The Kidney Disease Outcomes Quality Initiative (KDOQI) for Vascular Access recommends that the superficial venous segment chosen for arteriovenous fistula should have at least a 2.5 mm cross sectional venous diameter as shown on ultrasound, continuous with unobstructed proximal deep veins[8].

When evaluating superficial venous segments for venous puncture and cannulation, ultrasound analysis has been shown to increase success rates significantly with an odd ratio of 3.96 found on a meta-analysis that gathered various studies on successful venous puncture[14]. Adequate venous diameters vary from at least 2.0 to 2.5 mm [15,16], suggesting choice of an adequate superficial venous diameter similar to that needed for arteriovenous fistula construction.

Venous ultrasound guidance increases success rates for peripheral venous puncture as well as for arteriovenous fistula construction[13,14,17,18]. Its routine use requires human training and availability of ultrasound devices.

The search for and identification of clinical signs that would translate into finding a more reliable site for venous puncture or even for arteriovenous fistula construction may simplify these procedures, maintaining confidence and possibly reducing cost, though not replacing the gold standard which is vascular mapping with ultrasound.

Superficial venous percussion is a relatively understudied clinical sign. It was described at the end of 19 century as the Schwartz maneuver, in lower limbs, as a way to test for superficial venous insufficiency[19]. In this test, the mechanical wave produced through percussion on a proximal saphenous venous segment is transmitted distally if the saphenous vein is patent and its valves are insufficient. Considering this, percussion of superficial venous segments as cephalic and basilic veins in upper limbs would lead to a mechanical wave felt on proximal segments, if there were no significant obstruction or stenosis between the site where the wave is generated through distal tapping and where it is felt on a proximal segment. Once proven that the use of venous percussion in upper limbs superficial veins can correlate or translate into patent and adequate superficial venous segments in upper limbs, a new and not previously described use for this clinical sign, its use can lead to easier physical evaluation of patent superficial venous segments in the upper limbs.

## Objective

To assess the ability of venous percussion to check for patency and caliber adequacy of superficial veins of upper limbs.

## Methods

The present study was evaluated and approved by Rio Grande do Norte’s State University Ethics and Research Committee, Brazil, with Ethical Assessment certificate of presentation: 41865214.0.0000.5294. Participants signed the Written and Informed Consent Form.

Patients were recruited between May of 2015 and August of 2016.

This was a cross-sectional analytical study to compare upper limb venous percussion as a semiological sign, contrasted with venous ultrasound findings.

### Population and sample

The population sample was defined based on previous studies[20–22], corresponding to 70 individuals divided into two groups, A and B.

Group A consisted of 35 volunteers of both genders who required preoperative evaluation for arteriovenous fistula construction.

#### Inclusion criteria group A

Individuals who presented chronic kidney disease and were on preoperative evaluation for arteriovenous fistula construction.

#### Exclusion criteria group A

Individuals who presented a debilitating clinical condition that made it difficult to perform venous duplex ultrasound or complete physical examination, as well as those who refused to sign the Informed Consent Form, and those vulnerable and / or incapable.

Group B was composed of 35 volunteers of both genders, non-renal, selected by convenience in the ambulatory population who sought general medical attention at a primary clinic. There was no intention or protocol to match the patients in Group B with the ones in Group A for any demographic parameter. The main intention was to test the ability of venous percussion to check for patency and caliber adequacy of superficial veins of upper limbs in two completely different settings (Groups A and B).

#### Inclusion criteria group B

Individuals selected and invited by convenience in the ambulatory population who sought general medical attention at a primary clinic on specific days for patient recruitment.

#### Exclusion criteria group B

Individuals with symptoms related to the upper limbs or chronic kidney diseases; those who refused to sign the informed consent, as well as those vulnerable and / or incapacitated.

### Study variables

#### Characterization variables

The predictive variable consisted of a Positive or Negative response to venous percussion (transmission of mechanical wave). The response or dependent variable consisted of location and patency analyzed by venous duplex ultrasound, compressibility and non pulsatility of the vessel. This was assessed by measuring the diameters on 3 thirds of each of the venous segments of the main superficial draining veins of the upper limbs (cephalic and basilic veins), arm and forearm. Each measurement had to be equal to or greater than 2.5 millimeters. The absence of stenosis, phlebitis or thrombosis was also verified[8].

During physical examination, the maneuver described here was performed following the foregoing protocol, available at dx.doi.org/10.17504/protocols.io.4s7gwhn. The anatomical sites of the main four superficial venous segments of the upper limbs (cephalic vein in forearm, cephalic vein in arm, basilic vein in forearm and basilic vein in the arm) were struck distally with forefinger of the dominant hand with a tourniquet on the limb. Small impacts were generated on the patient's skin in the anatomical path of the superficial venous segment with the index finger of the dominant hand of the examiner at a point distal to the patient's limb, while the palmar face of the examiner's non-dominant hand was located at a proximal point along the way of this main superficial venous segment (about 15 cm away). In the non-dominant hand of the examiner (proximal), the proximal perception of the transmitted distal wave impacts caused by percussion (impacts) with the index finger of the dominant (distal) hand resulted in a positive maneuver on the venous segment examined. The maneuver was declared negative after at least 10 impacts generated by the dominant hand (distal) and not transmitted to the proximal hand of the examiner (about 15 seconds of attempts).

#### Initial assessments

The selected patients underwent a complete physical examination of, at least, one upper limb, preferably the non-dominant. In the non-renal group of patients, group B, the accuracy of the clinical signal was assessed by analysis of interobserver agreement, kappa index, among observers. This was accomplished by recording separately the impression of whether or not venous percussion was positive for the 35 patients in group B by a trained examiner (vascular surgeon) and examiner in training (fourth year medical school student in a six years program, with less than 1 hour of training for performing the maneuver). Curricular training had been previously provided on general physical examination and vascular examination (not including venous percussion) in the pertaining curricular disciplines. For the research, additional training was provided consisting of a simple explanation of the maneuver as well as a single demonstration on a healthy volunteer for each superficial venous segment on the upper limb (cephalic and basilic on both arm and forearm). Total additional training did not exceed 1 hour. No learning curve was measured.

Ultrasound was performed in B mode and with venous Doppler of the limbs studied, recording data on superficial vein caliber, non-pulsatility, thrombosis (compressibility or not) and parietal thickening in the B mode. The device used was either a Philips® HD7, Philips and Neusoft Medical Systems Co., Shenyang, China, with a linear transducer 3–12 MHz or a Toshiba® Xario, Canon Medical Systems Corporation, Barueri, Brazil, with linear transducer 5–11 MHz.

### Statistical analysis

Data was organized, categorized, and typed into Excel 2016 version 16.0, Microsoft Corporation, Redmond, USA. Statistical analysis was performed using the program Calc from LibreOffice version 5.3.7.2 (x64), The Document Foundation, Berlin, Germany, and the statistics program R version 3.4.2 (2017), The R Foundation, Vienna, Austria, using packages IswR 2.0–7, bootLR 1.0, epiR 0.9–93 and epibasix 1.3. The results were initially evaluated using a descriptive analysis, expressed by absolute numbers and percentages in categorical variables such as gender or presence of chronic diseases and by means and standard deviation (SD) for continuous variables such as age and Body Mass Index (BMI). The accuracy, sensitivity, specificity, true prevalence, estimated prevalence, positive predictive value and negative predictive value were calculated, as well as the 95% confidence interval (CI) of the results were obtained. The agreement between the methods, venous percussion and US with venous Doppler, were evaluated by the index kappa and LR (likelihood ratio) with 95% CIs. The agreement between observers measuring the signal venous percussion was evaluated by the kappa index with 95% CI’s. A Z test was performed to confirm if the p value <0.05 for Z test where H0 is kappa = or <0.21 (at least Fair or better agreement as measured by kappa value was statistically significant) for kappa measurements of agreement between methods (venous percussion and ultrasound) and between observers (medical student and vascular surgeon. Kappa index was interpreted accordingly to Viera & Garrett (2005) [23]as slight agreement, fair agreement, moderate agreement, substantial agreement and almost perfect agreement.

## Results

For each of the 70 participants (groups A and B) an upper limb was evaluated, preferably the non-dominant. In each upper limb evaluated, the results for venous percussion were recorded as well as superficial venous patency through duplex scan (B mode and Color Doppler). In such evaluation, the cephalic vein on the forearm and on the arm, as well as, the basilic vein on the forearm and on the arm were recorded. A total of 280 superficial venous upper limb segments were evaluated and recorded in both groups together: 70 cephalic veins on the forearm; 70 cephalic veins on the arm; 70 basilic veins on the forearm and 70 basilic veins on the arm. Thus, 140 segments of upper limb superficial veins were evaluated in each group.

Group A consisted of 35 individuals, 18 of whom were male and 17 female, with a mean of 56.03 (± 14.50 SD) years of age and 26.32 (± 5.75 SD) of Body Mass Index (BMI), all right-handed. Hypertension was present in 30 patients and diabetes mellitus in 19, but 17 presented both comorbidities and only 3 with none of them (Table 1).

**Table 1:** Demographic data, groups A and B.

	Mean Age	± SD	Sex		BMI							Chronic Diseases		
			F	M	Mean BMI	±SD	LW	NW	O	Obesity		DM	H	DM and H
										Class I	Class II			
**Group A**	56.03	14.50	17	18	26.32	5.75	1	14	11	5	4	19	30	17
**Group B**	42.57	12.58	23	12	27.35	5.02	0	14	8	10	3	0	6	0

BMI- Body Mass Index; F–Female; M–Male; LW–Low Weight (BMI < 18.5); NW–Normal Weight (BMI Between 18.5 and 24.9); O–Overweight (BMI Between 25 and 29.9); Obesity Class I (BMI Between 30 and 34.9); Obesity Class II (BMI > 35); DM–Diabetes Mellitus; H–Hypertension; SD–Standard Deviation.

In group A, the 19 diabetic patients had 25 venous segments (33%) adequate for venous duplex ultrasound criteria and 51 unsuitable segments (67%). The 16 non-diabetic patients had 15 adequate venous segments (23%) for venous duplex ultrasound and 49 unsuitable segments (77%). In this same group A, 15 patients presented BMI lower than 25; 20 patients had a BMI greater than 25. Patients with a BMI of less than 25 presented 10 adequate venous segments (17%) for duplex ultrasonography and 50 non-adequate segments (83%). On the other hand, the 20 patients with BMI greater than 25 presented 30 adequate venous segments (37%) for venous duplex scan and 50 non-adequate segments (63%).

Group B, in turn, consisted of 35 people, of whom 12 were male and 23 female, with a mean of 42.57 (± 12.58 SD) years of age and 27.35 (± 5.02 SD) of BMI, being 31 right-handed. No patient had diabetes mellitus; six were hypertensive.

In group B, 14 patients had a BMI of less than 25 and 21 patients had a BMI greater than 25. Patients with BMI <25 presented 56 adequate venous segments (61%) for duplex ultrasonography and 28 unsuitable segments (39%). On the other hand, the 21 patients with BMI greater than 25 presented 34 adequate venous segments (67%) and 22 non-adequate segments (33%).

As it was the intention of this research, and as shown by the demographic data, Groups A and B were constituted of two different populations, with a greater number of patients presenting Diabetes and Hypertension in Group A. As well, there was the predefined criteria of being or not a chronic renal disease patient needing hemodialysis for each group. The results found for each Group in this study represents the actual testing of percussion on two different populations (not a test-control) with a different prevalence of inadequate venous segments as will be demonstrated by the following results.

Interobserver agreement, a measure of precision of this new clinical sign which this study has named upper limb venous percussion, was assessed through comparative analysis above chance of agreement between an experienced examiner (vascular surgeon) and an inexperienced examiner (4-year medical student with a training of less than 1 hour in the execution of the maneuver). The kappa index presented global index for all venous segments of 0.741 (95% confidence interval-95% CI-0.632 to 0.851; a p value <0.001 for Z test where H0 is kappa = or <0.21).

**Fig 1:**
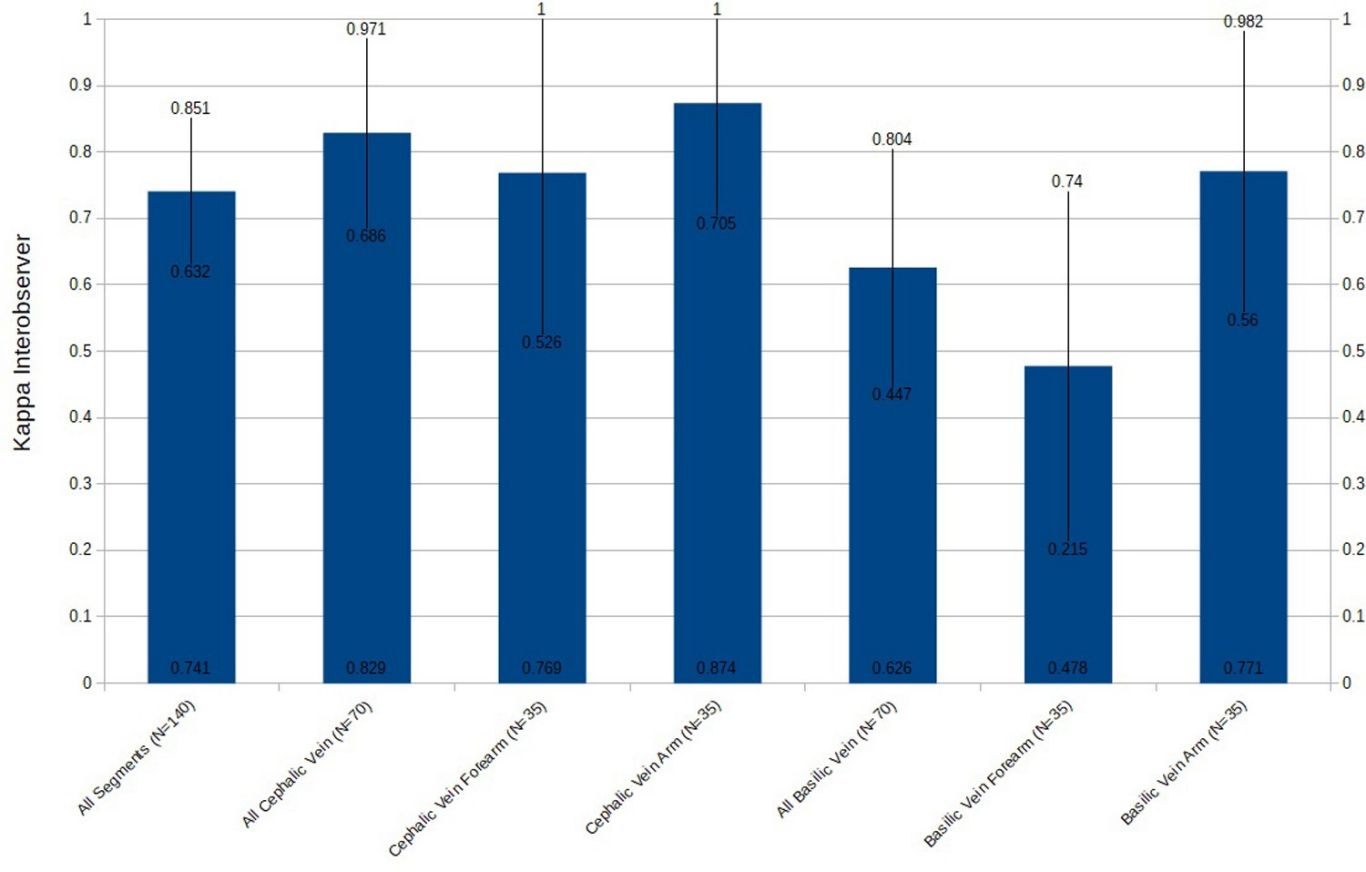
Kappa interobserver. In blue, Kappa index with p value <0.05, 95% CIs in vertical black lines.

The analysis of agreement between observers of the cephalic vein, as a whole, demonstrated a kappa index of 0.83 (95% CI 0.666 to 0.971; p value <0.001 for Z test where H0 is kappa = or <0.21). The basilic vein, as a whole, presented a kappa index of 0.63 (95% CI 0.477 to 0.804; p value <0.001 for Z test where H0 is kappa = or <0.21), Fig 1.

Of a total of 140 venous segments of group A, 101 were considered not adequate accordingly to the ultrasound criteria, because they presented one of their measurements (distal, middle or proximal) with a diameter smaller than 2.5 mm, that is, 72,1% inadequate venous segments in Group A. Out of these 101, inadequate venous segments identified by ultrasound, 97 (96%) had a negative percussion and 4 (4%) a positive percussion. Out of the total of 140 venous segments, 39 were considered appropriate (27,9%). However, the percussion was negative in 115 segments and positive in 25. Overall, when all venous segments were analyzed in group A, the kappa index of agreement between the percussion and the ultrasonography reached 0.56 (95% CI 0.401 to 0.72; p value <0.001 for Z test where H0 is kappa = or <0.21), a moderate agreement between the methods (Table 2). In all venous segments together in group A, the positive LR for percussion in compliance with the venous duplex ultrasound of the segment was 13.60 (CI 95% 5.95 to Infinite computed via BCa bootstrapping). The negative LR was 0.48 (95% CI 0.344 to 0.641 computed via BCa bootstrapping). In this group of renal patients, a positive venous percussion meant, through these results, at least a fivefold increase in the likelihood of such a venous segment being patent and having a continuous diameter of at least 2,5 mm on duplex ultrasound. On the other side, a negative venous percussion, in this group of renal patients, meant a decrease by approximately half (two fold decrease) in the likelihood of such a venous segment being patent and having a continuous diameter of at least 2,5 mm on duplex ultrasound.

**Table 2:** Evaluation of the agreement between venous percussion and ultrasound, according to the kappa index, sensitivity, specificity, true prevalence of adequate venous segments, estimated prevalence of adequate venous segments, positive predictive value, negative predictive value, accuracy, +LR and -LR in group A.

	Group A (N = 35 Patients)							
	Cephalic				Basilic			
	Forearm (N = 35)	CI 95%	Arm (N = 35)	CI 95%	Forearm (N = 35)	CI 95%	Arm (N = 35)	CI 95%
Sensitivity	1.00	0.63 ~ 1.00	0.77	0.46 ~ 0.95	0.50	0.01 ~ 0.99	0.13	0.02 ~ 0.38
Specificity	0.96	0.81 ~ 1.00	0.95	0.77 ~ 1.00	0.94	0.80 ~ 0.99	1.00	0.82 ~ 1.00
True Prevalence	0.23	0.10 ~ 0.40	0.37	0.21 ~ 0.55	0.06	0.01 ~ 0.19	0.46	0.29 ~ 0.63
Estimated Prevalence	0.26	0.12 ~ 0.43	0.31	0.17 ~ 0.49	0.09	0.02 ~ 0.23	0.06	0.01 ~ 0.19
Positive Predictive Value	0.89	0.52 ~ 1.00	0.91	0.59 ~ 1.00	0.33	0.01 ~ 0.91	1.00	0.16 ~ 1.00
Negative Predictive Value	1.00	0.87 ~ 1.00	0.88	0.68 ~ 0.97	0.97	0.84 ~ 1.00	0.58	0.39 ~ 0.75
Accuracy	0.97	0.85 ~ 1.0	0.89	0.73 ~ 0.97	0.91	0.77 ~ 0.98	0.60	0.42 ~ 0.76
Kappa Index	0.92***	0.77 ~ 1.07	0.75***	0.52 ~ 0.98	0.36 ^#^	-0.21 ~ 0.92	0.13^##^	-0.13 ~ 0.39
LR +	27	6.37 ~ ∞	16.92	3.75 ~ ∞	8.25	3.26 ~ ∞	∞	0.40 ~ ∞
LR -	0	0.00 ~ 0.32	0.24	0.00 ~ 0.49	0.53	0.00 ~ 1.11	0.87	0.68 ~ 1.05
Agreement[23]	Almost Perfect		Substantial		Fair		Slight	

*** = p value < 0.001;

^#^ = p value 0.29;

^##^ = p value 0.71;

CI = Confidence Interval; LR + = Positive Likelihood Ratio; LR— = Negative Likelihood Ratio.

In Group B, of the 140 total venous segments, 50 were inadequate (36,7%) and 90 adequate (64,3%) by ultrasound criteria. This represents more than two times the number of adequate venous segments of Group A, again demonstrating a higher prevalence of inadequate venous segments in Group A. From a total of 50 inadequate venous segments, 41 (82%) had negative percussion and 9 (18%) had positive percussion. The percussion was negative in 68 and positive in 72 segments, kappa index of 0.48, moderate agreement (95% CI 0.39 to 0.62; p value <0.001 for Z test where H0 is kappa = or <0.21). In all venous segments together, for group B, the positive LR for percussion in compliance with the venous duplex ultrasound of the segment was 3.89 (CI 95% 2.29 to 8.50 computed via BCa bootstrapping). The negative LR was 0.37 (CI 95% 0.25 to 0.50 computed via BCa bootstrapping).

As there is no interdependence between the results of each segment, forearm and arm, nor between the cephalic and basilic veins, consideration should be given to a separate analysis of each vein and each segment for better evaluation of venous percussion (Tables 2 and 3).

**Table 3:** Evaluation of the agreement between the venous percussion and the US, according to the kappa index, sensitivity, specificity, true prevalence of adequate venous segments, estimated prevalence of adequate venous segments, positive predictive value, negative predictive value, accuracy, +LR and -LR in group B.

	Group B (N = 35 Patients)							
	Cephalic				Basilic			
	Forearm (N = 35)	CI 95%	Arm (N = 35)	CI 95%	Forearm (N = 35)	CI 95%	Arm (N = 35)	CI 95%
Sensitivity	0.88	0.68 ~ 0.97	0.83	0.63 ~ 0.95	0.50	0.19 ~ 0.81	0.53	0.35 ~ 0.71
Specificity	0.64	0.31 ~ 0.89	0.82	0.48 ~ 0.98	0.88	0.69 ~ 0.97	1.00	0.29 ~ 1.00
True Prevalence	0.69	0.51 ~ 0.83	0.69	0.51 ~ 0.83	0.29	0.15 ~ 0.46	0.91	0.77 ~ 0.98
Estimated Prevalence	0.71	0.54 ~ 0.85	0.63	0.45 ~ 0.79	0.23	0.10 ~ 0.40)	0.49	0.31 ~ 0.66
Positive Predictive Value	0.84	0.64 ~ 0.95	0.91	0.71 ~ 0.99	0.63	0.24 ~ 0.91	1.00	0.80 ~ 1.00
Negative Predictive Value	0.70	0.35 ~ 0.93	0.69	0.39 ~ 0.91	0.81	0.62 ~ 0.94	0.17	0.04 ~ 0.41
Accuracy	0.80	0.63 ~ 0.91	0.83	0.66 ~ 0.93	0.77	0.60 ~ 0.89	0.57	0.39 ~ 0.74
Kappa Index	0.52*	0.22 ~ 0.83	0.62***	0.35 ~ 0.89	0.40^#^	0.06 ~ 0.74	0.16^##^	-0.08 ~ 0.41
LR +	2.41	1.31 ~ 9.95	4.58	1.84 ~ ∞	4.17	1.29 ~ ∞	∞	0.66 ~ ∞
LR -	0.20	0.04 ~ 0.48	0.20	0.04 ~ 0.44	0.57	0.21 ~ 0.92	0.47	1.73 ~ 0.33
Agreement[23]	Moderate		Substantial		Fair		Slight	

* = p value < 0.05;

*** = p value < 0.001;

^#^ = p value 0.114;

^##^ = p value 0.644;

LR + = Positive Likelihood Ratio; LR— = Negative Likelihood Ratio.

The results can be seen to be more favorable for the cephalic vein than for the basilic vein (Tables 2, 3 and 4). Agreement between methods in the cephalic vein ranged from substantial to almost perfect, with only one result of moderate agreement. It is important to emphasize that this vein is the most used for venous puncture and arteriovenous fistula construction. In addition, the high positive predictive value associated with high specificity corroborates the practical utility of a positive result to the percussion, with a great chance of success corresponding to vessel patency and adequate diameter.

**Table 4:** Evaluation of agreement between venous percussion and US, according to the Kappa index, Sensitivity, Specificity, True Prevalence of adequate venous segments, Estimated Prevalence of adequate venous segments, Positive Predictive Value, Negative Predictive Value, Accuracy, +LR and -LR, All venous segments Group A, All venous segments Group B.

	All Venous Segments Group A (N = 140)	CI 95%	All Venous Segments Group B (N = 140)	CI 95%
Sensitivity	0.54	0.37 ~ 0.70	0.70	0.59 ~ 0.79
Specificity	0.96	0.90 ~ 0.99	0.82	0.69 ~ 0.91
True Prevalence	0.28	0.21 ~ 0.36	0.64	0.56 ~ 0.72
Estimated Prevalence	0.18	0.12 ~ 0.25	0.51	0.43 ~ 0.60
Positive Predictive Value	0.84	0.64 ~ 0.95	0.88	0.78 ~ 0.94
Negative Predictive Value	0.84	0.76 ~ 0.90	0.60	0.48 ~ 0.72
Accuracy	0.84	0.77 ~ 0.90	0.74	0.66 ~ 0.81
Kappa Index	0.56***	0.40~ 0.72	0.48***	0.34~ 0.62
LR +	13.60	4.98 ~ 37.08	3.89	2.12 ~ 7.13
LR -	0.48	0.34 ~ 0.68	0.37	0.26 ~ 0.51
Agreement[23]	Moderate		Moderate	

*** = p value <0.001;

LR + = Positive Likelihood Ratio; LR— = Negative Likelihood Ratio.

The percussion of the cephalic vein in the forearm in dialysis patients in Group A presented the best results obtained in this study. There was only one percussion that did not correspond to the ultrasound result, characterizing a false positive result. Thus, the sensitivity reached 1.0 (95% CI 0.63 to 1.00) and specificity 0.96 (95% CI 0.81 to 1.00), with positive predictive values of 0.89 (CI 95% 0.52 to 1.00) and negative of 1.0 (95% CI 0.87 to 1.00). The kappa index was 0.92 (95% CI 0.77 to 1.00; p value <0.001 for Z test where H0 is kappa = or <0.21), which means an excellent agreement between methods, percussion and ultrasound. In group B, non renal, the kappa index resulted in 0.52 (95% CI 0.22 to 0.83; p value <0.001 for Z test where H0 is kappa = or <0.21), indicating a moderate agreement between the methods. However, it is important to consider that the prevalence was different in the groups, with more than twice the number of inadequate veins in group A.

## Discussion

The superficial venous physical exam for a peripheral venous puncture, or for the construction of an arteriovenous fistula does not include percussion as an auxiliary physical examination diagnostic method [8–11]. Comparing the results found, mainly in relation to the cephalic vein (preferential vein in both peripheral venous puncture and arteriovenous fistula construction), it is possible to infer that the inclusion of this maneuver could add important information to the clinical examination, with a degree of substantial to almost perfect agreement for duplex venous ultrasound criteria of adequacy, though not a replacement for duplex venous ultrasound. The criteria used for venous adequacy of ultrasound are the same, demonstrating a significant increase in the rate of success in a arteriovenous fistula construction or success rate in a peripheral venous puncture[13,16,20].

Before addressing the diagnostic accuracy of a test, it is important to analyze its precision. One way to measure the precision of the test is the kappa index, which translates agreement above chance of a test result between two different executors of the same test, regardless of whether the result with which they agree translates to a correct diagnosis or not[24]. In this study it was observed that the kappa index showed statistical significance (p value < 0.05) for the presence of at least a reasonable correlation in all segments of cephalic vein (renal or non renal) for segment adequacy by ultrasound criteria, the gold standard of evaluation, pointing to the value of this maneuver in physical examination. Since there is no similar description in the literature on the ability of venous percussion to diagnose an adequate superficial venous segment of upper limbs suitable for venous puncture or for arteriovenous fistula construction, a comparison of the results found in this study with previous studies of clinical signs in different clinical situations[25–32], found that the indices regarding traditional physical examinations techniques such as pulmonary auscultation were inferior to the ones found in this study[25]. This provides evidence for including venous percussion in a traditional physical examination, as part of the triage done during physical examination, and, maybe with further studies, on already existing scales for difficult venous access [3,33]. However, it is important to state clearly that in no way can venous percussion replace the outcomes provided by duplex ultrasound mapping.

A Receiver Operating Characteristic (ROC) curve was not included because the authors thought that a ROC curve analysis does not fit for the results of this research’s protocol, since the binary (positive or negative) result of venous percussion as compared to ultrasound (gold standard) is not decided by a numeric quantitative cutoff such as a score of a scale or a measurement of a bio marker as in other types of diagnostic methods. Therefore, there is no cutoff point to change and determine different numbers of sensitivity and specificity for the test at each cutoff point (what would be necessary for the essence of a ROC curve) [34,35]. Considering the ROC curve not as a two axis graph (sensitivity vs (1- specificity)), but as what it really is, a three axis measurement that depends on a hidden axis (the cutoff values), this diagnostic method, venous percussion, lacks the third axis of different cutoff values.

Analysis of the sensitivity, specificity and diagnostic accuracy of a clinical sign in a structured and systematic way enables identification of possible forms of assisting diagnosis of clinical conditions in a simple manner. From the point of view of public health, this can have a real impact on the logistics of diagnosis on remote areas of undeveloped countries as has been demonstrated in cases of community-acquired pneumonia in children[29,30,32].

In the study carried out in this research, the cephalic vein presented a sensitivity of 100% (95% CI 63% to 100%) and specificity of 96% (95% CI 81% to 100%) in the renal group identified by appropriate criteria of ultrasonography for venous puncture or arteriovenous fistula construction. It is important however to acknowledge the wide confidence interval in these results, especially regarding the sensitivity, which is a limitation of this study. The association of this clinical sign in the evaluation of possible venous puncture sites may also be helpful in remote locations where there is no access to auxiliary devices such as an ultrasound or a phleboscope, or in emergency situations where there is no time to bring in these devices. A previous survey among physicians concerning the use of ultrasound in central venous line placement in an advanced unit, such as intensive care in a developed country (United States), has reported limited availability of ultrasound equipment as a barrier for ultrasound use (28%) [36,37]. Even though, as ultrasound becomes more portable and reduces its cost, it tends to be more available for the physician, this is not yet the reality in a third word country as Brazil.

The costs related to the performance of guided peripheral venous puncture with ultrasound, besides the logistic aspects, have already been studied and are not low[38], especially considering that around 15% of peripheral venous accesses are considered difficult [3].

Failure in the first attempt at venous puncture increases pain scores[3]. Considering that peripheral venous puncture procedures are common, subject to difficulties that generate multiple attempts, and that these multiple attempts result in greater pain and morbidity to the patients involved, one can infer that it is in the best interest of the patients to identify those with potentially difficulty for peripheral venous puncture and direct these patients to ancillary methods such as ultrasound-guided peripheral venous puncture.

Up to the time of the present study, we were able to identify 2 studies of predictors of success (scales) for peripheral venous puncture in order to stratify patients more selectively for the use of associated technologies such as guided puncture by ultrasound or the use of a phleboscope. In 2014, Torre-Montero and colleagues published a study carried out with 56 oncology patients and 52 control patients, where the measurements of superficial venous calibers by ultrasound were crossed with the score that 3 observers performed based on three parameters (number of points of observable punctures, better catheter size for cannulation and ease of puncture / risk of extravasation). An excellent level of agreement among observers and a reduction in diameter proportional to the scores obtained on the scale was observed (Venous International Assessment—VIA scale)[33]. A problem in external application of these findings in clinical situations other than oncology patients is that the scoring parameters are generally based on the performances of punctures (number of puncture points observable, estimated ease for puncture / risk of extravasation) and less based on evaluations directly related to the physical examination of any patient. This fact suggests that perhaps, outside the cancer group where multiple punctures are common, the application of this scale as a predictor of failure in peripheral venous puncture may not fare so well. In 2016, a scale called A-DIVA (Adult Difficult Intra Venous Access scale) was developed based on a broad cohort of 1063 surgical patients punctured by certified anesthetists and nurse anesthetists[3]. On the A-DIVA scale the 5 factors identified from a list of 24 signs, symptoms or antecedents were: the presence of palpable veins; the presence of visible veins; a history of difficult access; unplanned surgery and venous diameter estimated to be less than 2.0 mm (measurement performed with a ruler)[3]. Patients were classified as low risk, medium risk and high risk for failure, with a sensitivity of 40% and specificity of 93% for failure at the first puncture in the high-risk for failure group[3]. The scale’s ability to identify success in the first puncture in the low risk for failure group showed a sensitivity of 85% for first puncture success and specificity of 80% for success in the first puncture[3]. Despite the use of a large cohort, with solid statistical analysis for the scale, the A-DIVA scale also focused on a specific clinical situation (surgical patients) and analyzed all the risk factors for unsuccessful puncture only carried out by individuals with considerable experience in peripheral venous puncture (anesthesiologists and anesthetist nurses). The question remains whether, with less experienced personnel and in different places other than a surgical center (such as in emergency room, pre-hospital or rural areas,) this scale would have a similar performance in predicting first puncture failure. As the venous percussion of upper limbs superficial veins had not yet been described before the present study, it was not included on this scale.

Considering arteriovenous fistulas, the site of first choice by surgeons is the wrist, performing anastomosis between the radial artery and the cephalic vein[8–11,39]. Described by Brescia and Cimino in 1962[40], the radio-cephalic AVF is recommended as the first choice for arteriovenous construction[8–11], although it only maturates in 55% of cases and has a 1 year patency of 65%[41], especially if either the artery or the vein used is inadequate[8,39]. The cephalic vein, for which the best agreement with the ultrasound was observed in this research, when used in a brachio-cephalic configuration, is also the one associated with the best outcomes in terms of a balance between patency and complications[41].

Venous percussion, mainly of the cephalic vein, has potential to assist in the anatomical location and evaluation of the quality of venous segments, both for venous puncture in the upper limbs and for choice of a venous segment for arteriovenous fistula construction in dialysis patients. Routine implementation of venous percussion evaluation in the assessment of superficial veins of the upper limbs in the clinical situations described above may have a logistic impact on health care.

## Conclusion

Venous percussion has high positive predictive value and high specificity in the evaluation of patency and adequacy of the superficial veins of the upper limbs, especially to check the patency and adequacy of the cephalic vein.

It is clear that venous percussion needs to be added to the traditional physical examination as it pertains to superficial venous evaluation of upper limbs. Although not the intention of this research, insufficient evidence was found to suggest it being able to replace duplex evaluation. It is important, however, that future studies on difficult venous access scales consider including this maneuver as one of the items being evaluated.

## Supporting information

S1 File The original file IC SENSESPvppvpn.doc with all titules in English. (DOC)
