# Supplementary material for: Evaluation of upper limb superficial venous percussion as a sign of anatomical location and venous permeability. A comparative study of superficial venous percussion to ultrasound findings on non-renal patients and on chronic kidney disease patients
Source: PLoS One. 2019 Nov 11;14(11):e0224825. doi: 10.1371/journal.pone.0224825 (PMC6844462; doi:10.1371/journal.pone.0224825)
Supplement: S1 File — (DOC) [file pone.0224825.s001.doc]

SENSITIVITY AND SPECIFICITY WITH CONFIDENCE LIMITS

APPARENT PREVALENCE

TRUE PREVALENCE

POSITIVE PREDICTIVE VALUE

NEGATIVE PREDICTIVE VALUE

POSITIVE LIKELIHOOD RATIO

NEGATIVE LIKELIHOOD RATIO

NON-RENAL

CEPHALIC AND BASILIC VEINS

> dat <- cbind(c(63,27), c(9,41))

> summary(sensSpec(dat))

Detailed Sensitivity and Specitivity Output

Input Matrix:

Gold Standard A Gold Standard B

Reported A 63 9

Reported B 27 41

The sample sensitivity is: 70%

95% Confidence Limits for true sensitivity are: [60.5, 79.5]

The sample of specificity is: 82%

95% Confidence Limits for true specificity are: [71.4, 92.6]

The sample value of Youden's J is: 52

95% Confidence Limits for Youden's J are: [37.8, 66.2]

Sample value for Percent Agreement (PA) is: 74.3%

95% Confidence Limits for PA are: [67, 81.5]

> dat <- as.table(matrix(c(63,9,27,41), nrow = 2, byrow = TRUE))

> colnames(dat) <- c("Usg+","Usg-")

> rownames(dat) <- c("Perc+","Perc-")

> rval <- epi.tests(dat, conf.level = 0.95)

> print(rval); summary(rval)

Outcome + Outcome - Total

Test + 63 9 72

Test - 27 41 68

Total 90 50 140

Point estimates and 95 % CIs:

---------------------------------------------------------

Apparent prevalence 0.51 (0.43, 0.60)

True prevalence 0.64 (0.56, 0.72)

Sensitivity 0.70 (0.59, 0.79)

Specificity 0.82 (0.69, 0.91)

Positive predictive value 0.88 (0.78, 0.94)

Negative predictive value 0.60 (0.48, 0.72)

Positive likelihood ratio 3.89 (2.12, 7.13)

Negative likelihood ratio 0.37 (0.26, 0.51)

---------------------------------------------------------

est lower upper

aprev 0.5142857 0.4284004 0.5995539

tprev 0.6428571 0.5575455 0.7219841

se 0.7000000 0.5942788 0.7921181

sp 0.8200000 0.6856306 0.9142379

diag.acc 0.7428571 0.6621982 0.8129489

diag.or 10.6296296 4.5398587 24.8882256

nnd 1.9230769 1.4157167 3.5725849

youden 0.5200000 0.2799094 0.7063560

ppv 0.8750000 0.7759281 0.9412167

npv 0.6029412 0.4769658 0.7196513

plr 3.8888889 2.1196535 7.1348722

nlr 0.3658537 0.2600759 0.5146532

NON-RENAL

CEPHALIC VEIN FOREARM

> dat <- cbind(c(21,3), c(4,7))

> summary(sensSpec(dat))

Detailed Sensitivity and Specitivity Output

Input Matrix:

Gold Standard A Gold Standard B

Reported A 21 4

Reported B 3 7

The sample sensitivity is: 87.5%

95% Confidence Limits for true sensitivity are: [74.3, 100.7]

The sample of specificity is: 63.6%

95% Confidence Limits for true specificity are: [35.2, 92.1]

The sample value of Youden's J is: 51.1

95% Confidence Limits for Youden's J are: [19.8, 82.5]

Sample value for Percent Agreement (PA) is: 80%

95% Confidence Limits for PA are: [66.7, 93.3]

> dat <- as.table(matrix(c(21,4,3,7), nrow = 2, byrow = TRUE))

> colnames(dat) <- c("Usg+","Usg-")

> rownames(dat) <- c("Perc+","Perc-")

> rval <- epi.tests(dat, conf.level = 0.95)

> print(rval); summary(rval)

Outcome + Outcome - Total

Test + 21 4 25

Test - 3 7 10

Total 24 11 35

Point estimates and 95 % CIs:

---------------------------------------------------------

Apparent prevalence 0.71 (0.54, 0.85)

True prevalence 0.69 (0.51, 0.83)

Sensitivity 0.88 (0.68, 0.97)

Specificity 0.64 (0.31, 0.89)

Positive predictive value 0.84 (0.64, 0.95)

Negative predictive value 0.70 (0.35, 0.93)

Positive likelihood ratio 2.41 (1.09, 5.34)

Negative likelihood ratio 0.20 (0.06, 0.62)

---------------------------------------------------------

est lower upper

aprev 0.7142857 0.53695536 0.8536453

tprev 0.6857143 0.50712000 0.8314828

se 0.8750000 0.67638864 0.9734407

sp 0.6363636 0.30790472 0.8907366

diag.acc 0.8000000 0.63062116 0.9155940

diag.or 12.2500000 2.18456193 68.6922617

nnd 1.9555556 -63.66732781 1.1571700

youden 0.5113636 -0.01570664 0.8641772

ppv 0.8400000 0.63917155 0.9546205

npv 0.7000000 0.34754715 0.9332605

plr 2.4062500 1.08526473 5.3351398

nlr 0.1964286 0.06226468 0.6196802

NON-RENAL

CEPHALIC VEIN ARM

> dat <- cbind(c(20,4), c(2,9))

> summary(sensSpec(dat))

Detailed Sensitivity and Specitivity Output

Input Matrix:

Gold Standard A Gold Standard B

Reported A 20 2

Reported B 4 9

The sample sensitivity is: 83.3%

95% Confidence Limits for true sensitivity are: [68.4, 98.2]

The sample of specificity is: 81.8%

95% Confidence Limits for true specificity are: [59, 104.6]

The sample value of Youden's J is: 65.2

95% Confidence Limits for Youden's J are: [37.9, 92.4]

Sample value for Percent Agreement (PA) is: 82.9%

95% Confidence Limits for PA are: [70.4, 95.3]

> dat <- as.table(matrix(c(20,2,4,9), nrow = 2, byrow = TRUE))

> colnames(dat) <- c("Usg+","Usg-")

> rownames(dat) <- c("Perc+","Perc-")

> rval <- epi.tests(dat, conf.level = 0.95)

> print(rval); summary(rval)

Outcome + Outcome - Total

Test + 20 2 22

Test - 4 9 13

Total 24 11 35

Point estimates and 95 % CIs:

---------------------------------------------------------

Apparent prevalence 0.63 (0.45, 0.79)

True prevalence 0.69 (0.51, 0.83)

Sensitivity 0.83 (0.63, 0.95)

Specificity 0.82 (0.48, 0.98)

Positive predictive value 0.91 (0.71, 0.99)

Negative predictive value 0.69 (0.39, 0.91)

Positive likelihood ratio 4.58 (1.29, 16.26)

Negative likelihood ratio 0.20 (0.08, 0.52)

---------------------------------------------------------

est lower upper

aprev 0.6285714 0.44923116 0.7852679

tprev 0.6857143 0.50712000 0.8314828

se 0.8333333 0.62615829 0.9526464

sp 0.8181818 0.48224415 0.9771688

diag.acc 0.8285714 0.66350170 0.9343782

diag.or 22.5000000 3.46491373 146.1075339

nnd 1.5348837 1.07548255 9.2248851

youden 0.6515152 0.10840243 0.9298152

ppv 0.9090909 0.70838726 0.9887944

npv 0.6923077 0.38573834 0.9090796

plr 4.5833333 1.29191499 16.2603148

nlr 0.2037037 0.07981396 0.5198990

NON-RENAL

BASILIC VEIN FOREARM

> dat <- cbind(c(5,5), c(3,22))

> summary(sensSpec(dat))

Detailed Sensitivity and Specitivity Output

Input Matrix:

Gold Standard A Gold Standard B

Reported A 5 3

Reported B 5 22

The sample sensitivity is: 50%

95% Confidence Limits for true sensitivity are: [19, 81]

The sample of specificity is: 88%

95% Confidence Limits for true specificity are: [75.3, 100.7]

The sample value of Youden's J is: 38

95% Confidence Limits for Youden's J are: [4.5, 71.5]

Sample value for Percent Agreement (PA) is: 77.1%

95% Confidence Limits for PA are: [63.2, 91.1]

> dat <- as.table(matrix(c(5,3,5,22), nrow = 2, byrow = TRUE))

> colnames(dat) <- c("Usg+","Usg-")

> rownames(dat) <- c("Perc+","Perc-")

> rval <- epi.tests(dat, conf.level = 0.95)

> print(rval); summary(rval)

Outcome + Outcome - Total

Test + 5 3 8

Test - 5 22 27

Total 10 25 35

Point estimates and 95 % CIs:

---------------------------------------------------------

Apparent prevalence 0.23 (0.10, 0.40)

True prevalence 0.29 (0.15, 0.46)

Sensitivity 0.50 (0.19, 0.81)

Specificity 0.88 (0.69, 0.97)

Positive predictive value 0.62 (0.24, 0.91)

Negative predictive value 0.81 (0.62, 0.94)

Positive likelihood ratio 4.17 (1.22, 14.24)

Negative likelihood ratio 0.57 (0.30, 1.07)

---------------------------------------------------------

est lower upper

aprev 0.2285714 0.1042104 0.4013633

tprev 0.2857143 0.1463547 0.4630446

se 0.5000000 0.1870860 0.8129140

sp 0.8800000 0.6878097 0.9745346

diag.acc 0.7714286 0.5986367 0.8957896

diag.or 7.3333333 1.3005436 41.3502299

nnd 2.6315789 -7.9933317 1.2699242

youden 0.3800000 -0.1251043 0.7874486

ppv 0.6250000 0.2448632 0.9147666

npv 0.8148148 0.6191701 0.9370000

plr 4.1666667 1.2188400 14.2439620

nlr 0.5681818 0.3006562 1.0737533

NON-RENAL

BASILIC VEIN ARM

> dat <- cbind(c(17,15), c(0,3))

> summary(sensSpec(dat))

Detailed Sensitivity and Specitivity Output

Input Matrix:

Gold Standard A Gold Standard B

Reported A 17 0

Reported B 15 3

The sample sensitivity is: 53.1%

95% Confidence Limits for true sensitivity are: [35.8, 70.4]

The sample of specificity is: 100%

95% Confidence Limits for true specificity are: [100, 100]

The sample value of Youden's J is: 53.1

95% Confidence Limits for Youden's J are: [35.8, 70.4]

Sample value for Percent Agreement (PA) is: 57.1%

95% Confidence Limits for PA are: [40.7, 73.5]

> dat <- as.table(matrix(c(17,0,15,3), nrow = 2, byrow = TRUE))

> colnames(dat) <- c("Usg+","Usg-")

> rownames(dat) <- c("Perc+","Perc-")

> rval <- epi.tests(dat, conf.level = 0.95)

> print(rval); summary(rval)

Outcome + Outcome - Total

Test + 17 0 17

Test - 15 3 18

Total 32 3 35

Point estimates and 95 % CIs:

---------------------------------------------------------

Apparent prevalence 0.49 (0.31, 0.66)

True prevalence 0.91 (0.77, 0.98)

Sensitivity 0.53 (0.35, 0.71)

Specificity 1.00 (0.29, 1.00)

Positive predictive value 1.00 (0.80, 1.00)

Negative predictive value 0.17 (0.04, 0.41)

Positive likelihood ratio Inf (NaN, Inf)

Negative likelihood ratio 0.47 (0.32, 0.68)

---------------------------------------------------------

est lower upper

aprev 0.4857143 0.31382851 0.6601086

tprev 0.9142857 0.76942498 0.9819624

se 0.5312500 0.34743681 0.7090602

sp 1.0000000 0.29240177 1.0000000

diag.acc 0.5714286 0.39353094 0.7367728

diag.or Inf NaN Inf

nnd 1.8823529 -2.77653284 1.4103175

youden 0.5312500 -0.36016142 0.7090602

ppv 1.0000000 0.80493568 1.0000000

npv 0.1666667 0.03578508 0.4141775

plr Inf NaN Inf

nlr 0.4687500 0.32415369 0.6778469

CHRONIC KIDNEY DISEASE PATIENTS

CEPHALIC AND BASILIC VEINS

> dat <- cbind(c(21,18), c(4,97))

> summary(sensSpec(dat))

Detailed Sensitivity and Specitivity Output

Input Matrix:

Gold Standard A Gold Standard B

Reported A 21 4

Reported B 18 97

The sample sensitivity is: 53.8%

95% Confidence Limits for true sensitivity are: [38.2, 69.5]

The sample of specificity is: 96%

95% Confidence Limits for true specificity are: [92.2, 99.8]

The sample value of Youden's J is: 49.9

95% Confidence Limits for Youden's J are: [33.8, 66]

Sample value for Percent Agreement (PA) is: 84.3%

95% Confidence Limits for PA are: [78.3, 90.3]

> dat <- as.table(matrix(c(21,4,18,97), nrow = 2, byrow = TRUE))

> colnames(dat) <- c("Usg+","Usg-")

> rownames(dat) <- c("Perc+","Perc-")

> rval <- epi.tests(dat, conf.level = 0.95)

> print(rval); summary(rval)

Outcome + Outcome - Total

Test + 21 4 25

Test - 18 97 115

Total 39 101 140

Point estimates and 95 % CIs:

---------------------------------------------------------

Apparent prevalence 0.18 (0.12, 0.25)

True prevalence 0.28 (0.21, 0.36)

Sensitivity 0.54 (0.37, 0.70)

Specificity 0.96 (0.90, 0.99)

Positive predictive value 0.84 (0.64, 0.95)

Negative predictive value 0.84 (0.76, 0.90)

Positive likelihood ratio 13.60 (4.98, 37.08)

Negative likelihood ratio 0.48 (0.34, 0.68)

---------------------------------------------------------

est lower upper

aprev 0.1785714 0.1190223 0.2522182

tprev 0.2785714 0.2061804 0.3605834

se 0.5384615 0.3718107 0.6990522

sp 0.9603960 0.9016950 0.9891055

diag.acc 0.8428571 0.7718052 0.8988217

diag.or 28.2916667 8.6790526 92.2241681

nnd 2.0045802 1.4531553 3.6562305

youden 0.4988576 0.2735057 0.6881577

ppv 0.8400000 0.6391715 0.9546205

npv 0.8434783 0.7639540 0.9045165

plr 13.5961538 4.9849012 37.0830622

nlr 0.4805710 0.3416127 0.6760535

CHRONIC KIDNEY DISEASE PATIENTS

CEPHALIC VEIN FOREARM

> dat <- cbind(c(8,0), c(1,26))

> summary(sensSpec(dat))

Detailed Sensitivity and Specitivity Output

Input Matrix:

Gold Standard A Gold Standard B

Reported A 8 1

Reported B 0 26

The sample sensitivity is: 100%

95% Confidence Limits for true sensitivity are: [100, 100]

The sample of specificity is: 96.3%

95% Confidence Limits for true specificity are: [89.2, 103.4]

The sample value of Youden's J is: 96.3

95% Confidence Limits for Youden's J are: [89.2, 103.4]

Sample value for Percent Agreement (PA) is: 97.1%

95% Confidence Limits for PA are: [91.6, 102.7]

> dat <- as.table(matrix(c(8,1,0,26), nrow = 2, byrow = TRUE))

> colnames(dat) <- c("Usg+","Usg-")

> rownames(dat) <- c("Perc+","Perc-")

> rval <- epi.tests(dat, conf.level = 0.95)

> print(rval); summary(rval)

Outcome + Outcome - Total

Test + 8 1 9

Test - 0 26 26

Total 8 27 35

Point estimates and 95 % CIs:

---------------------------------------------------------

Apparent prevalence 0.26 (0.12, 0.43)

True prevalence 0.23 (0.10, 0.40)

Sensitivity 1.00 (0.63, 1.00)

Specificity 0.96 (0.81, 1.00)

Positive predictive value 0.89 (0.52, 1.00)

Negative predictive value 1.00 (0.87, 1.00)

Positive likelihood ratio 27.00 (3.95, 184.78)

Negative likelihood ratio 0.00 (0.00, NaN)

---------------------------------------------------------

est lower upper

aprev 0.2571429 0.1248940 0.4325588

tprev 0.2285714 0.1042104 0.4013633

se 1.0000000 0.6305834 1.0000000

sp 0.9629630 0.8102944 0.9990627

diag.acc 0.9714286 0.8508279 0.9992769

diag.or Inf NaN Inf

nnd 1.0384615 1.0009381 2.2682025

youden 0.9629630 0.4408777 0.9990627

ppv 0.8888889 0.5175035 0.9971909

npv 1.0000000 0.8677254 1.0000000

plr 27.0000000 3.9452446 184.7794181

nlr 0.0000000 0.0000000 NaN

CHRONIC KIDNEY DISEASE PATIENTS

CEPHALIC VEIN ARM

> dat <- cbind(c(10,3), c(1,21))

> summary(sensSpec(dat))

Detailed Sensitivity and Specitivity Output

Input Matrix:

Gold Standard A Gold Standard B

Reported A 10 1

Reported B 3 21

The sample sensitivity is: 76.9%

95% Confidence Limits for true sensitivity are: [54, 99.8]

The sample of specificity is: 95.5%

95% Confidence Limits for true specificity are: [86.8, 104.2]

The sample value of Youden's J is: 72.4

95% Confidence Limits for Youden's J are: [47.9, 96.9]

Sample value for Percent Agreement (PA) is: 88.6%

95% Confidence Limits for PA are: [78, 99.1]

> dat <- as.table(matrix(c(10,1,3,21), nrow = 2, byrow = TRUE))

> colnames(dat) <- c("Usg+","Usg-")

> rownames(dat) <- c("Perc+","Perc-")

> rval <- epi.tests(dat, conf.level = 0.95)

> print(rval); summary(rval)

Outcome + Outcome - Total

Test + 10 1 11

Test - 3 21 24

Total 13 22 35

Point estimates and 95 % CIs:

---------------------------------------------------------

Apparent prevalence 0.31 (0.17, 0.49)

True prevalence 0.37 (0.21, 0.55)

Sensitivity 0.77 (0.46, 0.95)

Specificity 0.95 (0.77, 1.00)

Positive predictive value 0.91 (0.59, 1.00)

Negative predictive value 0.88 (0.68, 0.97)

Positive likelihood ratio 16.92 (2.44, 117.52)

Negative likelihood ratio 0.24 (0.09, 0.65)

---------------------------------------------------------

est lower upper

aprev 0.3142857 0.16851715 0.4928800

tprev 0.3714286 0.21473212 0.5507688

se 0.7692308 0.46186846 0.9496189

sp 0.9545455 0.77155560 0.9988499

diag.acc 0.8857143 0.73262196 0.9679688

diag.or 70.0000000 6.44515759 760.2606971

nnd 1.3816425 1.05433096 4.2840485

youden 0.7237762 0.23342406 0.9484688

ppv 0.9090909 0.58722008 0.9977010

npv 0.8750000 0.67638864 0.9734407

plr 16.9230769 2.43700042 117.5176378

nlr 0.2417582 0.08923664 0.6549669

CHRONIC KIDNEY DISEASE PATIENTS

BASILIC VEIN FOREARM

> dat <- cbind(c(1,1), c(2,31))

> summary(sensSpec(dat))

Detailed Sensitivity and Specitivity Output

Input Matrix:

Gold Standard A Gold Standard B

Reported A 1 2

Reported B 1 31

The sample sensitivity is: 50%

95% Confidence Limits for true sensitivity are: [-19.3, 119.3]

The sample of specificity is: 93.9%

95% Confidence Limits for true specificity are: [85.8, 102.1]

The sample value of Youden's J is: 43.9

95% Confidence Limits for Youden's J are: [-25.8, 113.7]

Sample value for Percent Agreement (PA) is: 91.4%

95% Confidence Limits for PA are: [82.2, 100.7]

> dat <- as.table(matrix(c(1,2,1,31), nrow = 2, byrow = TRUE))

> colnames(dat) <- c("Usg+","Usg-")

> rownames(dat) <- c("Perc+","Perc-")

> rval <- epi.tests(dat, conf.level = 0.95)

> print(rval); summary(rval)

Outcome + Outcome - Total

Test + 1 2 3

Test - 1 31 32

Total 2 33 35

Point estimates and 95 % CIs:

---------------------------------------------------------

Apparent prevalence 0.09 (0.02, 0.23)

True prevalence 0.06 (0.01, 0.19)

Sensitivity 0.50 (0.01, 0.99)

Specificity 0.94 (0.80, 0.99)

Positive predictive value 0.33 (0.01, 0.91)

Negative predictive value 0.97 (0.84, 1.00)

Positive likelihood ratio 8.25 (1.20, 56.84)

Negative likelihood ratio 0.53 (0.13, 2.13)

---------------------------------------------------------

est lower upper

aprev 0.08571429 0.018037640 0.2305750

tprev 0.05714286 0.006996764 0.1915714

se 0.50000000 0.012579117 0.9874209

sp 0.93939394 0.797735687 0.9925742

diag.acc 0.91428571 0.769424981 0.9819624

diag.or 15.50000000 0.685186343 350.6345425

nnd 2.27586207 -5.271892715 1.0204133

youden 0.43939394 -0.189685195 0.9799950

ppv 0.33333333 0.008403759 0.9057007

npv 0.96875000 0.837829006 0.9992091

plr 8.25000000 1.197425802 56.8406827

nlr 0.53225806 0.132756654 2.1339695

CHRONIC KIDNEY DISEASE PATIENTS

BASILIC VEIN ARM

> dat <- cbind(c(2,14), c(0,19))

> summary(sensSpec(dat))

Detailed Sensitivity and Specitivity Output

Input Matrix:

Gold Standard A Gold Standard B

Reported A 2 0

Reported B 14 19

The sample sensitivity is: 12.5%

95% Confidence Limits for true sensitivity are: [-3.7, 28.7]

The sample of specificity is: 100%

95% Confidence Limits for true specificity are: [100, 100]

The sample value of Youden's J is: 12.5

95% Confidence Limits for Youden's J are: [-3.7, 28.7]

Sample value for Percent Agreement (PA) is: 60%

95% Confidence Limits for PA are: [43.8, 76.2]

> dat <- as.table(matrix(c(2,0,14,19), nrow = 2, byrow = TRUE))

> colnames(dat) <- c("Usg+","Usg-")

> rownames(dat) <- c("Perc+","Perc-")

> rval <- epi.tests(dat, conf.level = 0.95)

> print(rval); summary(rval)

Outcome + Outcome - Total

Test + 2 0 2

Test - 14 19 33

Total 16 19 35

Point estimates and 95 % CIs:

---------------------------------------------------------

Apparent prevalence 0.06 (0.01, 0.19)

True prevalence 0.46 (0.29, 0.63)

Sensitivity 0.12 (0.02, 0.38)

Specificity 1.00 (0.82, 1.00)

Positive predictive value 1.00 (0.16, 1.00)

Negative predictive value 0.58 (0.39, 0.75)

Positive likelihood ratio Inf (NaN, Inf)

Negative likelihood ratio 0.88 (0.73, 1.05)

---------------------------------------------------------

est lower upper

aprev 0.05714286 0.006996764 0.1915714

tprev 0.45714286 0.288271444 0.6335420

se 0.12500000 0.015513604 0.3834762

sp 1.00000000 0.823533088 1.0000000

diag.acc 0.60000000 0.421117722 0.7612919

diag.or Inf NaN Inf

nnd 8.00000000 -6.212981966 2.6077235

youden 0.12500000 -0.160953308 0.3834762

ppv 1.00000000 0.158113883 1.0000000

npv 0.57575758 0.392153016 0.7452382

plr Inf NaN Inf

nlr 0.87500000 0.727071410 1.0530259

CHRONIC KIDNEY DISEASE PATIENTS AND NON-RENAL PATIENTS

CEPHALIC AND BASILIC VEINS

> dat <- cbind(c(84,45), c(13,138))

> summary(sensSpec(dat))

Detailed Sensitivity and Specitivity Output

Input Matrix:

Gold Standard A Gold Standard B

Reported A 84 13

Reported B 45 138

The sample sensitivity is: 65.1%

95% Confidence Limits for true sensitivity are: [56.9, 73.3]

The sample of specificity is: 91.4%

95% Confidence Limits for true specificity are: [86.9, 95.9]

The sample value of Youden's J is: 56.5

95% Confidence Limits for Youden's J are: [47.1, 65.9]

Sample value for Percent Agreement (PA) is: 79.3%

95% Confidence Limits for PA are: [74.5, 84]

| > dat <- as.table(matrix(c(84,13,45,138), nrow = 2, byrow = TRUE))  > colnames(dat) <- c("Usg+","Usg-")  > rownames(dat) <- c("Perc+","Perc-")  > rval <- epi.tests(dat, conf.level = 0.95)  > print(rval); summary(rval)  Outcome + Outcome - Total  Test + 84 13 97  Test - 45 138 183  Total 129 151 280  Point estimates and 95 % CIs:  ---------------------------------------------------------  Apparent prevalence 0.35 (0.29, 0.41)  True prevalence 0.46 (0.40, 0.52)  Sensitivity 0.65 (0.56, 0.73)  Specificity 0.91 (0.86, 0.95)  Positive predictive value 0.87 (0.78, 0.93)  Negative predictive value 0.75 (0.69, 0.81)  Positive likelihood ratio 7.56 (4.43, 12.91)  Negative likelihood ratio 0.38 (0.30, 0.49)  ---------------------------------------------------------  est lower upper  aprev 0.3464286 0.2907989 0.4053484  tprev 0.4607143 0.4012338 0.5210350  se 0.6511628 0.5622970 0.7329148  sp 0.9139073 0.8572785 0.9533613  diag.acc 0.7928571 0.7406111 0.8387865  diag.or 19.8153846 10.0980642 38.8836376  nnd 1.7696920 1.4571395 2.3833608  youden 0.5650701 0.4195756 0.6862761  ppv 0.8659794 0.7817291 0.9266663  npv 0.7540984 0.6851414 0.8146310  plr 7.5635063 4.4306053 12.9116956  nlr 0.3816987 0.3000154 0.4856214 |
| --- |
| | > | | --- | |
|  |

dat <- as.table(matrix(c(29,5,3,33), nrow = 2, byrow = TRUE))

colnames(dat) <- c("Usg+","Usg-")

rownames(dat) <- c("Perc+","Perc-")

rval <- epi.tests(dat, conf.level = 0.95)

print(rval); summary(rval)

CHRONIC KIDNEY DISEASE PATIENTS AND NON-RENAL PATIENTS

CEPHALIC VEIN FOREARM

> dat <- as.table(matrix(c(29,5,3,33), nrow = 2, byrow = TRUE))

> colnames(dat) <- c("Usg+","Usg-")

> rownames(dat) <- c("Perc+","Perc-")

> rval <- epi.tests(dat, conf.level = 0.95)

> print(rval); summary(rval)

Outcome + Outcome - Total

Test + 29 5 34

Test - 3 33 36

Total 32 38 70

Point estimates and 95 % CIs:

---------------------------------------------------------

Apparent prevalence 0.49 (0.36, 0.61)

True prevalence 0.46 (0.34, 0.58)

Sensitivity 0.91 (0.75, 0.98)

Specificity 0.87 (0.72, 0.96)

Positive predictive value 0.85 (0.69, 0.95)

Negative predictive value 0.92 (0.78, 0.98)

Positive likelihood ratio 6.89 (3.02, 15.71)

Negative likelihood ratio 0.11 (0.04, 0.32)

---------------------------------------------------------

est lower upper

aprev 0.4857143 0.36440215 0.6082732

tprev 0.4571429 0.33744770 0.5805791

se 0.9062500 0.74977305 0.9802328

sp 0.8684211 0.71913615 0.9558626

diag.acc 0.8857143 0.78717196 0.9493478

diag.or 63.8000000 14.01254611 290.4853956

nnd 1.2908705 1.06826721 2.1326090

youden 0.7746711 0.46890920 0.9360954

ppv 0.8529412 0.68943427 0.9504715

npv 0.9166667 0.77531024 0.9824735

plr 6.8875000 3.02018457 15.7068733

nlr 0.1079545 0.03650272 0.3192689

CHRONIC KIDNEY DISEASE PATIENTS AND NON-RENAL PATIENTS

CEPHALIC VEIN ARM

> dat <- as.table(matrix(c(30,3,7,30), nrow = 2, byrow = TRUE))

> colnames(dat) <- c("Usg+","Usg-")

> rownames(dat) <- c("Perc+","Perc-")

> rval <- epi.tests(dat, conf.level = 0.95)

> print(rval); summary(rval)

Outcome + Outcome - Total

Test + 30 3 33

Test - 7 30 37

Total 37 33 70

Point estimates and 95 % CIs:

---------------------------------------------------------

Apparent prevalence 0.47 (0.35, 0.59)

True prevalence 0.53 (0.41, 0.65)

Sensitivity 0.81 (0.65, 0.92)

Specificity 0.91 (0.76, 0.98)

Positive predictive value 0.91 (0.76, 0.98)

Negative predictive value 0.81 (0.65, 0.92)

Positive likelihood ratio 8.92 (3.00, 26.53)

Negative likelihood ratio 0.21 (0.11, 0.41)

---------------------------------------------------------

est lower upper

aprev 0.4714286 0.3508785 0.5944724

tprev 0.5285714 0.4055276 0.6491215

se 0.8108108 0.6484476 0.9203789

sp 0.9090909 0.7566836 0.9808451

diag.acc 0.8571429 0.7529318 0.9293095

diag.or 42.8571429 10.1127156 181.6262582

nnd 1.3890785 1.1096021 2.4683358

youden 0.7199017 0.4051313 0.9012239

ppv 0.9090909 0.7566836 0.9808451

npv 0.8108108 0.6484476 0.9203789

plr 8.9189189 2.9984073 26.5297898

nlr 0.2081081 0.1058833 0.4090255

CHRONIC KIDNEY DISEASE PATIENTS AND NON-RENAL PATIENTS

BASILIC VEIN FOREARM

> dat <- as.table(matrix(c(6,5,6,53), nrow = 2, byrow = TRUE))

> colnames(dat) <- c("Usg+","Usg-")

> rownames(dat) <- c("Perc+","Perc-")

> rval <- epi.tests(dat, conf.level = 0.95)

> print(rval); summary(rval)

Outcome + Outcome - Total

Test + 6 5 11

Test - 6 53 59

Total 12 58 70

Point estimates and 95 % CIs:

---------------------------------------------------------

Apparent prevalence 0.16 (0.08, 0.26)

True prevalence 0.17 (0.09, 0.28)

Sensitivity 0.50 (0.21, 0.79)

Specificity 0.91 (0.81, 0.97)

Positive predictive value 0.55 (0.23, 0.83)

Negative predictive value 0.90 (0.79, 0.96)

Positive likelihood ratio 5.80 (2.11, 15.94)

Negative likelihood ratio 0.55 (0.31, 0.97)

---------------------------------------------------------

est lower upper

aprev 0.1571429 0.08114376 0.2637998

tprev 0.1714286 0.09184221 0.2803084

se 0.5000000 0.21094464 0.7890554

sp 0.9137931 0.81017404 0.9714140

diag.acc 0.8428571 0.73620023 0.9188562

diag.or 10.6000000 2.47044909 45.4816091

nnd 2.4166667 1.31497745 47.3514448

youden 0.4137931 0.02111868 0.7604693

ppv 0.5454545 0.23379360 0.8325119

npv 0.8983051 0.79168045 0.9617555

plr 5.8000000 2.11029575 15.9408936

nlr 0.5471698 0.30904018 0.9687893

CHRONIC KIDNEY DISEASE PATIENTS AND NON-RENAL PATIENTS

BASILIC VEIN ARM

> dat <- as.table(matrix(c(19,0,29,22), nrow = 2, byrow = TRUE))

> colnames(dat) <- c("Usg+","Usg-")

> rownames(dat) <- c("Perc+","Perc-")

> rval <- epi.tests(dat, conf.level = 0.95)

> print(rval); summary(rval)

Outcome + Outcome - Total

Test + 19 0 19

Test - 29 22 51

Total 48 22 70

Point estimates and 95 % CIs:

---------------------------------------------------------

Apparent prevalence 0.27 (0.17, 0.39)

True prevalence 0.69 (0.56, 0.79)

Sensitivity 0.40 (0.26, 0.55)

Specificity 1.00 (0.85, 1.00)

Positive predictive value 1.00 (0.82, 1.00)

Negative predictive value 0.43 (0.29, 0.58)

Positive likelihood ratio Inf (NaN, Inf)

Negative likelihood ratio 0.60 (0.48, 0.76)

---------------------------------------------------------

est lower upper

aprev 0.2714286 0.1719912 0.3909798

tprev 0.6857143 0.5636670 0.7914702

se 0.3958333 0.2576990 0.5473042

sp 1.0000000 0.8456275 1.0000000

diag.acc 0.5857143 0.4616616 0.7022790

diag.or Inf NaN Inf

nnd 2.5263158 1.8271375 9.6780631

youden 0.3958333 0.1033265 0.5473042

ppv 1.0000000 0.8235331 1.0000000

npv 0.4313725 0.2934549 0.5775468

plr Inf NaN Inf

nlr 0.6041667 0.4805186 0.7596320
